# Parameter Estimation and Prediction of COVID-19 Epidemic Turning Point and Ending Time of a Case Study on SIR/SQAIR Epidemic Models

**DOI:** 10.1155/2020/1465923

**Published:** 2020-12-27

**Authors:** Amir Hossein Amiri Mehra, Mohsen Shafieirad, Zohreh Abbasi, Iman Zamani

**Affiliations:** ^1^Department of Electrical and Computer Engineering, University of Kashan, Kashan, Iran; ^2^Electrical and Electronic Engineering Department, Shahed University, Tehran, Iran

## Abstract

In this paper, the SIR epidemiological model for the COVID-19 with unknown parameters is considered in the first strategy. Three curves (*S*, *I*, and *R*) are fitted to the real data of South Korea, based on a detailed analysis of the actual data of South Korea, taken from the Korea Disease Control and Prevention Agency (KDCA). Using the least square method and minimizing the error between the fitted curve and the actual data, unknown parameters, like the transmission rate, recovery rate, and mortality rate, are estimated. The goodness of fit model is investigated with two criteria (SSE and RMSE), and the uncertainty range of the estimated parameters is also presented. Also, using the obtained determined model, the possible ending time and the turning point of the COVID-19 outbreak in the United States are predicted. Due to the lack of treatment and vaccine, in the next strategy, a new group called quarantined people is added to the proposed model. Also, a hidden state, including asymptomatic individuals, which is very common in COVID-19, is considered to make the model more realistic and closer to the real world. Then, the SIR model is developed into the SQAIR model. The delay in the recovery of the infected person is also considered as an unknown parameter. Like the previous steps, the possible ending time and the turning point in the United States are predicted. The model obtained in each strategy for South Korea is compared with the actual data from KDCA to prove the accuracy of the estimation of the parameters.

## 1. Introduction and Problem Statement

The novel coronavirus (COVID-19) is an emerging disease that was first spread from Wuhan, China. This disease has developed in the entire world and has attracted worldwide attention. Since COVID-19 has affected more than 200 countries worldwide, it is important to model this disease correctly and identify how it spreads and predict the disease to take the necessary steps. In this regard, predictive mathematical models play a crucial role in investigating the epidemic spreading in a lack of specific antivirals or effective vaccine. Many articles have been published about disease modeling and estimating the unknown parameters of infectious diseases, including COVID-19. Hence, in the following, after reviewing the other papers, we will discuss our objectives and innovations in the present paper.

Accordingly, the authors in [1] estimated the parameters of the SIR model of COVID-19 in India using an actual data set. Also, Bastos and Cajueiro [2] have used two variations of the SIR-type model (SIR and SIAS) to forecast the evolution of the SARS-CoV-2 virus with the real data in Brazil. The second wave forecasting of spreading the COVID-19 in Iran with the SIR model is considered in [3]. The authors in [4] have also forecasted the trend of COVID-19 using the least square error (LSE) technique. Furthermore, in [5], the key epidemic parameters are estimated in the generalized SEIR model to forecast COVID-19 on epidemic size, peak time, and possible ending time for five different regions. The asymptomatic and quarantined people have not been considered in these papers. Due to the nature of COVID-19, it is recommended to consider the group of quarantined and asymptomatic individuals. Therefore, in our work, we modified the SIR model by considering the asymptomatic people, and people were put into quarantine. Also, the author in [6] introduced a SIR-type model that only considered the asymptomatic individuals for COVID-19 in Northern Italy based on parameter estimation.

In [7], the parameters and initial values of the SIR epidemic model are estimated for reported case data of the Hong Kong seasonal influenza epidemic in New York City in 1968-1969, to recognize the relevance between unreported and reported cases. The study by Liu et al. [8] is aimed at developing the mathematical model considering a new group of unreported cases for the COVID-19 epidemic in Wuhan, in which the parameters and the initial conditions of the proposed model are estimated. Thereupon, using the parameterized model, the number of unreported cases is identified. Hence, since the initial values are known in the present study, obtained through real data, there is no requirement to estimate them. The study by Hadeler [9] is aimed at identifying the time-dependent transmission rate in epidemic SIR, SIRS, and SEIRS models and reviewing and comparing the various results. In addition, in [10], the authors estimate the infection rate *β* of a SIR epidemic model based on input-output (IO) equations depending on the known quantity of output measurement and its derivatives. Furthermore, the authors in [11] introduced a more complete epidemic model for influenza that can be used for other diseases by parameter modification. In this regard, the authors in [12] have applied optimal control to the proposed epidemic model for COVID-19 compared to Ebola and influenza.

There are many different methods to estimate parameters in various epidemic models that can be used as required. For example, if new data is added during the identification process, then the model should be based on the observations until the current time. Therefore, the parameter estimation should be computed recursively over time, as described in [13] in detail. Moreover, if the model is considered two-dimensional, the study by Shafieirad et al. [14] can be helpful. In addition to the continuous models considered for epidemic dynamics, discrete models can also be used, which are discussed in [15] in detail. Also, since some people who have previously been infected with COVID-19 have been reported to be resusceptible, the authors in [16] introduced a modified SEIRS model considering the possibility of susceptibility for recovered people for control action. In [17], a new mathematical model with time-dependent coefficients is used to characterize the dynamics of COVID-19 in three countries: S. Korea, Italy, and Brazil.

Since the prevalence of COVID-19 in the United States is on the rise, it is vital to make predictions on the possible ending time. The method mentioned in this article can be applied to other countries and similar diseases. Since the prevalence of COVID-19 in South Korea has decreased and there is a complete set of data, taken from KDCA, an accurate model can be obtained to predict the ending point of the disease in other countries (including the United States).

Our motivation is to evaluate our method's efficiency on a classical SIR and SQAIR epidemiological model to predict the turning point and ending time of the COVID-19 disease in the United States. For this purpose, the method used in this study is the following.

Using actual data of South Korea, taken from KDCA, which has provided accurate and well-documented statistics on the prevalence of the coronavirus disease, the epidemic model's unknown parameters can be estimated. Using the obtained determined model, the possible ending time of COVID-19 in the United States can be predicted. Also, we use two strategies in this article to implement our motivation:

In the first strategy, the unknown parameters of a classical SIR (susceptible-infected-recovered) epidemiological model are estimated using the LS method more easily. Therefore, the turning point and ending time of COVID-19 in the United States are predicted. There may be asymptomatic carriers in the community in the incubation period despite having the disease and even despite the COVID-19 test result leading to transmit the disease to others. As a result, in this study, we also considered this group of people as asymptomatic people in our model and modified the basic SIR model to the SAIR model. Additionally, since there is no cure or vaccine for COVID-19 yet, it is necessary to quarantine susceptible people, and since there are no groups to include this group (*Q*) in the SAIR model, therefore in the next step, the SAIR model is developed to the SQAIR model by introducing a quarantine group. Besides, considering the delay in transferring people from the infected group to the recovered group is an essential factor added to the SQAIR model because it makes the model more realistic and closer to the natural process of spreading COVID-19. The same steps are then applied to the SQAIR model to achieve the turning point and ending time of the COVID-19 outbreak in the United States. Since there is no proper viral treatment or effective vaccine yet to prevent and control the spreading rate, currently, the best options and widely used strategies for decreasing the outbreak's growth rate are social distancing, stay-at-home orders, self-quarantine, lockdowns, isolation, and wearing a face mask.

In this paper, the group of quarantined people (*Q*) refers to all the above strategies, which are only called quarantined people, for this group's simplicity. As mentioned in the papers above, other groups can be added to the model, but this study is aimed at predicting COVID-19 with a comprehensive and straightforward model to incorporate the general features of the COVID-19 disease and can easily express the behavior of the disease. Furthermore, considering the delay in systems is critical because it is closer to the real world. Hence, in this study, delay in transferring infected people to the group of recovered people is considered an unknown parameter.

In the following, the general structure of the paper is presented.

In the first section of the paper, the introduction and problem statement were introduced. Our paper continues with Section 2, which presents the SIR model with dynamic equations and diagrams. In Section 3, the estimation of unknown parameters, model upgrade, prediction, and comparing results are presented. Finally, the conclusion is given in Section 4.

## 2. The SIR Epidemic Model

The SIR epidemic model used in this paper is described as follows: let *S* be the number of susceptible people to infection, *I* the number of infected people (people who have been tested positive for COVID-19), and *R* the number of recovered people. The SIR epidemic model is given by

(1a) $$\begin{array}{c} S_{k+1}=-\beta S_{k}I_{k}, \end{array}$$

(1b) $$\begin{array}{c} I_{k+1}=\beta S_{k}I_{k}-\left(g+\mu_{d}\right)I_{k}, \end{array}$$

(1c) $$\begin{array}{c} R_{k+1}=gI_{k}, \end{array}$$

(2) $$\begin{array}{c} N=S_{k}+I_{k}+R_{k}, \end{array}$$

where *β* is the transmission rate and the initial conditions are *S*_0_ ≥ 0, *I*_0_ ≥ 0, and *R*_0_ ≥ 0. All states are positive values (*S*, *I*, *R* ≥ 0). The total population *N* includes individuals who have been tested. In other words, the total number of people considered as a statistical population (due to the normalization, *N*) is equal to one.

**Remark 1 .**

The total population *N* includes the individuals who have been tested (it is a statistical society that can be generalized to the total size of the population), which is generally variable. But in this work, it is fixed and equal to the total number of people on the last day that data is taken (72nd day). According to the other researches, *N* is usually considered as the whole number of the country population; however, it is challenging to consider all population sizes of the people (almost 330 million in the U.S.) involved with COVID-19 because this disease is not equally distributed in all the states of a vast country like the United States. Hence, we considered a smaller community (people who have been tested) as our statistical society, which contains all three groups of people (*S*, *I*, and *R*). The parameters are also more accurately identified. For example, suppose the number of infected people is 500,000 and the total number of population is about 330 million. In that case, the ratio of infected people to the total population becomes small, and the estimated parameters are not obtained correctly. Of course, when we consider the statistical society as tested individuals, we can generalize them to the entire population.

**Figure 1:**
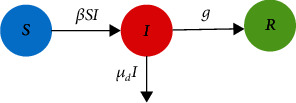
The conceptual flow diagram of the SIR dynamic model.

As shown in Figure 1, the infected people recover at a rate of *g*. *μ*_*d*_ indicates the removal rate of infected people due to mortality caused by infection.

Since the epidemic model parameters are unknown, estimating these parameters with the real data taken from South Korea is the main objective of this paper. As a result, using the known parameters, the spread of infection in the United States can be predicted by the method presented in Section 3.

**Remark 2 .**

The nature of epidemic models is discrete because data are collected and/or reported over discrete units of time that makes it easier to compare data with the output of a discrete model and can be easily implemented. For system identification, it is required to measure the input and output data in the time domain. Then, select a model structure (usually discrete model) and apply an estimation method (LS method in this paper) to estimate unknown parameter values. Since, in this study, the identification data, taken from medical reports, are daily, the discrete desired model structure is determined. Furthermore, these data may be weekly (also daily) in fast-spreading epidemics, such as influenza, SARS, Ebola, and especially novel coronavirus (COVID-19). Basically, epidemic modeling is all discrete in nature which can be considered continuous with a small step length. Of course, after estimating the parameters, it can be simply written in continuous form. Furthermore, the numerical investigation of discrete-time epidemic models is more straightforward. There has been some study of discrete epidemic models referred to in our paper [15].

**Remark 3 .**

Assume that the initial values *S*_*k*_0__ ≥ 0, *I*_*k*_0__ ≥ 0, and *R*_*k*_0__ ≥ 0 in which *k*_0_ = 0 and all parameters (*μ*_*d*_, *β*, *g*) are all positive. In the mentioned model, the change rate of the susceptible people is as *S*_*k*+1_ = −*βS*_*k*_*I*_*k*_, which shows that susceptible people become infected with the rate of *β* and move from group *S* to *I*. Then, after a period when the number of susceptible people reaches zero, the rate of change (*S*_*k*+1_ = −*βS*_*k*_*I*_*k*_) becomes zero and remains unchanged. After the number of the susceptible people reaches zero on a specific day (*S*_*k*_1__ = 0), Eq. (1 − *b*) changes to *I*_*k*+1_ = −(*g* + *μ*_*d*_)*I*_*k*_ which is a difference equation that eventually tends to zero (*I*_*k*_2__ = 0). On the other hand, the number of recovered people increases at the rate of *g* and when the number of infected people reaches zero the recovered people remains at its maximum value (*R*_*k*_2__ = *R*_*m*_,  *R*_*m*_ > 0). As a result, on day *k*_2_(*k*_2_ > *k*_1_), we reach a stable equilibrium point (0, 0, *R*_*m*_), where *R*_*m*_ = *g*∑_*k*=0_^*k*_2_^*I*_*k*_.

## 3. Parameter Estimation, Prediction, and Comparing Results

According to the daily official reports of the Korea Disease Control and Prevention Agency (KDCA), the numbers of infected and daily deaths are available in public. The number of infected people (*I*) (people who have tested positive for COVID-19) and people who have died of the coronavirus disease (*d*) are specified in Table 1. Using Equations (1a)–(1c) and (2), the number of the susceptible (*S*) and recovered people can be computed

(3) $$\begin{array}{c} R_{k}=R_{k-1}+\left|I_{k}-I_{k-1}\right|-d_{k}, \end{array}$$

(4) $$\begin{array}{c} S_{k}=N-I_{k}-R_{k}. \end{array}$$

According to Equations (3) and (4), the number of infected, susceptible, and recovered people is determined. Minimizing an objective function leads to estimate the unknown parameters (*β*, *g*, *μ*_*d*_), presented in two strategies.

### 3.1. SIR Strategy

In the first strategy, three curves (*S*, *I*, and *R*) are fitted to the real data of South Korea, given in Table 1. The goodness of fit describes how well the function fits a set of actual data shown in Table 2 with two criteria, sum of square error (SSE) and root mean squared error (RMSE) that measure the deviation of the actual data from the curve fitted to the data. For these two criteria, the smaller the value, the better the model fits. Therefore, according to Table 2, the fit results are reasonable because the SSE and RMSE values are small and close to zero. Applying the least square method to the objective functions leads to estimate the unknown values of the parameters. The error between the fitted curves and the actual data is considered as the objective function. Given the objective functions *J*_1_, *J*_2_, and *J*_3_,

(5a) $$\begin{array}{c} J_{1}\left(\theta_{1}\right)=\frac{1}{N_{T}}\overset{N_{T}}{\underset{k=1}{\sum }}{{e_{S}}_{k}}^{2}, \end{array}$$

(5b) $$\begin{array}{c} J_{2}\left(\theta_{2}\right)=\frac{1}{N_{T}}\overset{N_{T}}{\underset{k=1}{\sum }}{{e_{I}}_{k}}^{2}, \end{array}$$

(5c) $$\begin{array}{c} J_{3}\left(\theta_{3}\right)=\frac{1}{N_{T}}\overset{N_{T}}{\underset{k=1}{\sum }}{{e_{R}}_{k}}^{2}, \end{array}$$

where *N*_*T*_ is the total number of data.

(6a) $$\begin{array}{c} {e_{S}}_{k}=\left({S_{\text{fit}}}_{k}-{\hat{S}}_{k}\right), \end{array}$$

(6b) $$\begin{array}{c} {e_{I}}_{k}=\left({I_{\text{fit}}}_{k}-{\hat{I}}_{k}\right), \end{array}$$

(6c) $$\begin{array}{c} {e_{R}}_{k}=\left({R_{\text{fit}}}_{k}-{\hat{R}}_{k}\right), \end{array}$$

in which *S*_fit__*k*_ is the number of susceptible individuals (in every *k*) obtained from the fitted curve to the actual data. The actual number of susceptible people is indicated by ${\hat{S}}_{k}={\phi_{1}}_{k}^{T}\theta_{1}$, where *ϕ*_1__*k*_^*T*^ = [−*S*_*k*_*I*_*k*_] and *θ*_1_ = [*β*]. Similarly, ${\hat{I}}_{k}={\phi_{2}}_{k}^{T}\theta_{2}$, in which *ϕ*_2__*k*_^*T*^ = [*S*_*k*_*I*_*k*_  − *I*_*k*_  − *I*_*k*_] and $\theta_{2}={\left[\begin{array}{ccc} \beta & g & \mu_{d} \end{array}\right]}^{T}$. Also, ${\hat{R}}_{k}={\phi_{3}}_{k}^{T}\theta_{3}$, where *ϕ*_3__*k*_^*T*^ = [*I*_*k*_] and *θ*_3_ = [*g*]. Finally, the optimal vectors (*θ*_1_, *θ*_2_, and *θ*_3_) are obtained using the least square method. Therefore,

(7a) $$\begin{array}{c} \theta_{1}={\left(\Phi_{1}^{T}\Phi_{1}\right)}^{-1}\Phi_{1}^{T}S, \end{array}$$

(7b) $$\begin{array}{c} \theta_{2}={\left(\Phi_{2}^{T}\Phi_{2}\right)}^{-1}\Phi_{2}^{T}I, \end{array}$$

(7c) $$\begin{array}{c} \theta_{3}={\left(\Phi_{3}^{T}\Phi_{3}\right)}^{-1}\Phi_{3}^{T}R, \end{array}$$

where $S={\left[\begin{array}{ccc} {S_{\text{fit}}}_{1} & {S_{\text{fit}}}_{2}\cdots & {S_{\text{fit}}}_{N_{T}} \end{array}\right]}^{T}$ in which, $\Phi_{1}={\left[\begin{array}{ccc} \phi_{1_{1}}^{T} & \phi_{1_{2}}^{T} & \phi_{1_{N_{T}}}^{T} \end{array}\right]}^{T}$ and $I={\left[\begin{array}{ccc} {I_{\text{fit}}}_{1} & {I_{\text{fit}}}_{2}\cdots & {I_{\text{fit}}}_{N_{T}} \end{array}\right]}^{T}$, $\Phi_{2}={\left[\begin{array}{ccc} \phi_{2_{1}}^{T} & \phi_{2_{2}}^{T} & \phi_{2_{N_{T}}}^{T} \end{array}\right]}^{T}$, $R={\left[\begin{array}{ccc} {R_{\text{fit}}}_{1} & {R_{\text{fit}}}_{2}\cdots & {R_{\text{fit}}}_{N_{T}} \end{array}\right]}^{T}$, and $\Phi_{3}={\left[\begin{array}{ccc} \phi_{3_{1}}^{T} & \phi_{3_{2}}^{T} & \phi_{3_{N_{T}}}^{T} \end{array}\right]}^{T}$.

Note that curve fitting is applied to the real data, based on a detailed analysis of the actual data of South Korea in Table 1, as mentioned. Then, using the least square method and minimizing the error between the fitted curve and the actual data, unknown parameters, like the transmission rate, recovery rate, and mortality rate (*β*, *g*, *μ*_*d*_), were obtained. Also, the uncertainty range of the estimated parameters is presented in Table 3.

**Figure 2:**
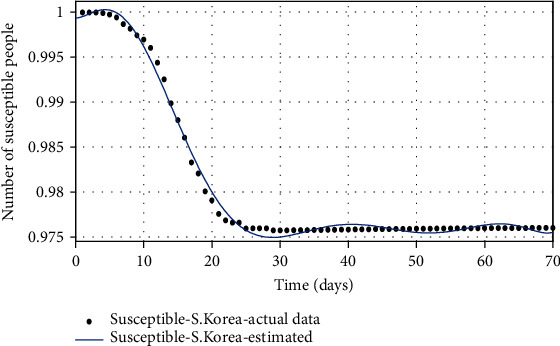
The comparison of the number of susceptible individuals based on actual data and the model obtained from the estimated parameters.

**Figure 3:**
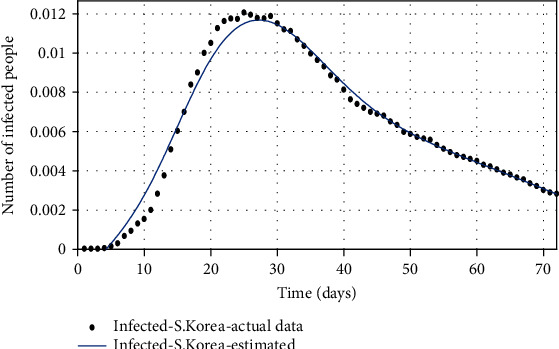
The comparison of the number of infected individuals based on actual data and the model obtained from the estimated parameters.

**Figure 4:**
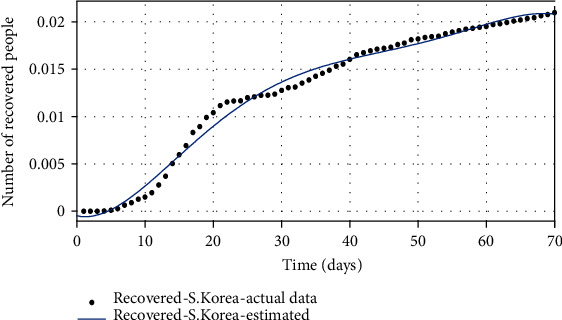
The comparison of the number of recovered individuals based on actual data and the model obtained from the estimated parameters.

The basic reproduction number can also be estimated as *R*_0_ = *β*/(*g* + *μ*_*d*_), based on estimated parameters (see [11] for details). The authors in [21] also estimated the reproduction number based on publicly available sources, which is a critical point in the outbreak of COVID-19, to investigate the growth rate of the COVID-19 outbreak in South Korea. According to Table 2, the uncertainty range of the basic reproduction number can be calculated in the following: *R*_0_min__ = *β*_min_/(*g*_max_ + *μ*_*d*__max_) as the lower range and *R*_0_max__ = *β*_max_/(*g*_min_ + *μ*_*d*__min_) as the upper range. The desired basic reproduction number can be calculated *R*_0_desired__ = *β*_mean_/(*g*_mean_ + *μ*_*d*__mean_) using the mean of parameters in Table 3. Then, the number of susceptible, infected, and recovered people is shown, respectively, in Figures 2–4. The real data series of the susceptible, infected, and recovered people obtained from Table 1 is compared with the number of people taken from the model with estimated parameters.

**Figure 5:**
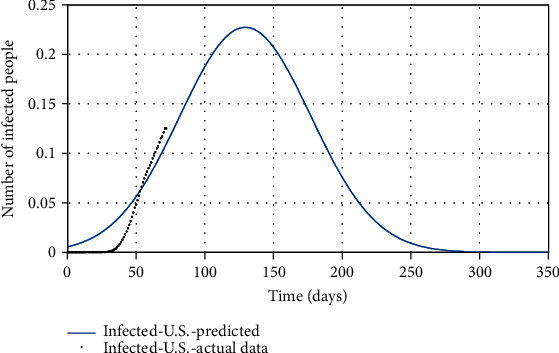
The actual number of infected people compared with the predicted number of them.

**Figure 6:**
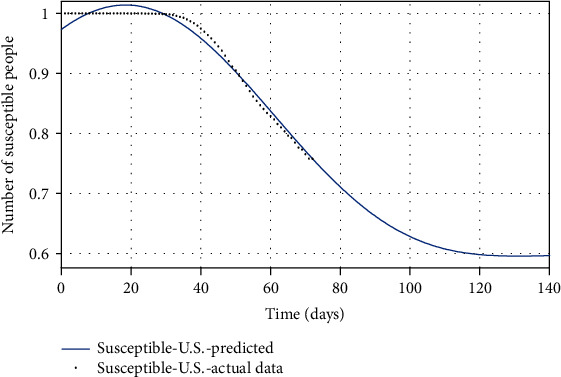
The actual number of susceptible people compared with the predicted number of them.

**Figure 7:**
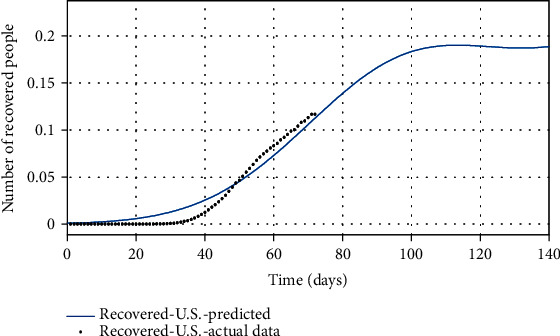
The actual number of recovered people compared with the predicted number of them.

As it turns out, the resulting SIR model is properly fitted to South Korean data, so this model can be used to predict the possible ending point of COVID-19 in the United States. Because COVID-19 is spreading out rapidly in the United States, it can be crucial to know the turning (inflection) point and possible ending time of the disease to make an effective decision. As shown in Figures 5–7, in the simplest strategy (SIR), the epidemic situation for the United States is not hopeful for the next 50 days, and the turning point of the disease is in the middle of June, and the number of infected people in the peak is about twice its current value (Apr. 28, 2020). However, fortunately, it is expected to end up completely within seven months (from Apr. 28, 2020).

However, in order to get closer to the real world, the model can be developed. Therefore, our studies will be expanded in the following strategy.

### 3.2. SQAIR Strategy

**Figure 8:**
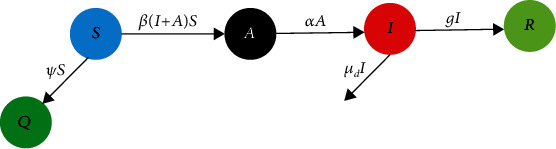
The conceptual flow diagram of the SQAIR dynamic model.

Since coronavirus disease is currently incurable, quarantine is a priority in all countries. Therefore, a new group called quarantined people can be added to the proposed model. Also, considering a new hidden state can make the model more realistic. This hidden state can be indicated by *A* that includes asymptomatic people, which is very common in COVID-19. Delay in the transfer of infected people to the group of recovered people is also considered. So, these three different conditions can be considered as follows:

1. The new group added (*A*) is infected people who have negative COVID-19 test and no symptoms. They are in their incubation period that can transmit the disease to others without any visible symptoms
2. In coronavirus disease, infected people continue to be carriers of the virus after recovery, so they remain in the infected group because they can continue to infect susceptible individuals at the rate of *β*, so they go to the group of recovered people with a delay (*k*_*d*_)
3. The quarantined people are shown by (*Q*).  Figure 8 shows the quarantine group and how to transfer to that group. In different countries, the quarantine rate of susceptible individuals may vary, so we consider this rate equal to *ψ*

Equations (1a)–(1c) are reformulated as follows:

(8a) $$\begin{array}{c} S_{k+1}=-\beta \left(I_{k}+A_{k}\right)S_{k}-\psi \;S_{k}, \end{array}$$

(8b) $$\begin{array}{c} Q_{k+1}=\psi \;S_{k}, \end{array}$$

(8c) $$\begin{array}{c} A_{k+1}=\beta \left(I_{k}+A_{k}\right)S_{k}-\alpha A_{k}, \end{array}$$

(8d) $$\begin{array}{c} I_{k+1}=\alpha A_{k}-\mu_{d}I_{k}-gI_{k-k_{d}}, \end{array}$$

(8e) $$\begin{array}{c} R_{k+1}=gI_{k-k_{d}}, \end{array}$$

where *α* is the rate of transfer of individuals from group *A* to *I*. The values of vectors *k*_*d*_ and *A*_*k*_ are unknown, and *ψ* is the quarantine rate. Since two new groups have been added to the model, the total number also changes.

**Remark 4 .**

Assume that the initial values *S*_*k*_0__ ≥ 0, *Q*_*k*_0__ ≥ 0, *A*_*k*_0__ ≥ 0, *I*_*k*_0__ ≥ 0, and *R*_*k*_0__ ≥ 0 in which *k*_0_ = 0 and all parameters (*μ*_*d*_, *β*, *g*, *ψ*, *α*) are all positive. The rate of change of the susceptible people is as *S*_*k*+1_ = −*ZS*_*k*_ in which *Z* = *β*(*I*_*k*_ + *A*_*k*_) + *ψ* ≥ 0, which remains at zero after zeroing the number of susceptible people (*S*_*k*_1__ = 0). The rate of change of *Q* is ascending, which remains at its maximum with the zeroing of susceptible individuals (*Q*_*k*_1__ = *Q*_*m*_). Then, after a period when the number of susceptible people reaches zero, the rate of change of asymptomatic people (*A*_*k*+1_ = *β*(*I*_*k*_ + *A*_*k*_)*S*_*k*_ − *αA*_*k*_) becomes *A*_*k*+1_ = −*αA*_*k*_ which is a difference equation that eventually tends to zero (*A*_*k*_2__ = 0, *k*_2_ > *k*_1_). After day *k*_2_, the rate of changes in the infected people changes as *I*_*k*+1_ = −*μ*_*d*_*I*_*k*_ − *gI*_*k*−*k*_*d*__ which has a downward trend and converges to zero (*I*_*k*_3__ = 0, *k*_3_ > *k*_2_ > *k*_1_), and according to *R*_*k*+1_ = *gI*_*k*−*k*_*d*__, the recovered people reach its maximum value on day *k*_3_ and remains stationary (*R*_*k*_3__ = *R*_*m*_). As a result, the stable equilibrium point of the model is obtained as (0, *Q*_*m*_, 0, 0, *R*_*m*_), where *Q*_*m*_ = *ψ*∑_*k*=0_^*k*_1_^*S*_*k*_ and *R*_*m*_ = *g*∑_*k*=*k*_*d*__^*k*_3_^*I*_*k*_.

First, it is assumed that there is no group of quarantined people (*Q*) and the rate *α* is not affected by quarantined people; therefore,

(9) $$\begin{array}{c} N^{\prime }=S_{k}+I_{k}+A_{k}+R_{k}. \end{array}$$

The total population *N*′ includes the individuals who have tested (it is a statistical society that can be generalized to the total size of the population), which is generally variable. But in this work, the total population is fixed and equal to the total number of people on the last day that data was taken (72nd day). According to Equation (9), the number of *I*_*k*_ and *R*_*k*_ is obtained from the actual data directly. Since *N*′ is known, the number of *A*_*k*_ + *S*_*k*_ can be calculated. There is no reported data for the number of *A* alone. Since the normalized value of *N*′ is equal to one and the number of infected and recovered people for South Korea is known, therefore

(10) $$\begin{array}{c} A_{k}+S_{k}=1-I_{k}-R_{k}. \end{array}$$

**Figure 9:**
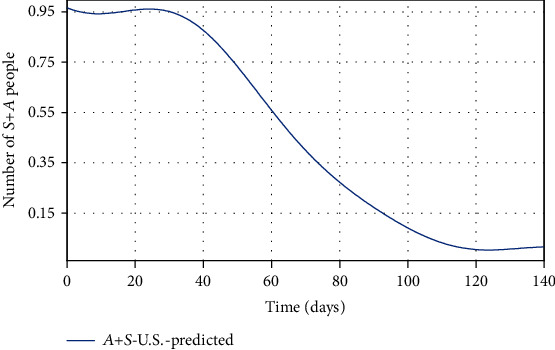
The total predicted number of two groups (*A* and *S*).

Due to the incubation period of COVID-19, it is difficult to separate these two groups, and as shown in Figure 9, considering the quarantine rate of 95%, it is predicted that the total number of susceptible and asymptomatic people in the United States will eventually reach almost zero in two months. Since the number of each state is positive, then the sum of them is positive too. If the number of *A* + *S* reaches zero, then the number of *A* and *S* must become zero individually. But even though we do not know the number of asymptomatic people, but in the end, we are sure that they will reach zero. Using the actual data in Table 1, a function (or curve) is fitted for the vector *A*_*k*_ + *S*_*k*_. Similarly, as mentioned before, the goodness of fit model is investigated with two criteria in Table 4. According to Table 4, small SSE and RMSE indicate a close fit of the function to the data. Therefore, our model fits very encouraging based on South Korean data.

Then, by derivation from the obtained function and equating it with Equation (11) that is obtained by Equations (8a) and (8c), an unknown value *α* can be obtained.

(11) $$\begin{array}{c} S_{k+1}+A_{k+1}=-\alpha A_{k}. \end{array}$$

Now to make the parameters more accurate and to choose the optimal parameter, similar to the previous one, using the LS method

(12) $$\begin{array}{c} J_{4}\left(\theta_{4}\right)=\frac{1}{N_{T}}\overset{N_{T}}{\underset{k=1}{\sum }}{{e_{\left(S+A\right)}}_{k}}^{2}, \end{array}$$

where ${e_{\left(S+A\right)}}_{k}=\left({{\left(S+A\right)}_{\text{fit}}}_{k}-{\left(\hat{S+A}\right)}_{k}\right)$, in which (*S* + *A*)_fit__*k*_ is the total number of *S* and *A* obtained from the fitted curve. Besides that, ${\left(\hat{S+A}\right)}_{k}={\phi_{4}}_{k}^{T}\theta_{4}$ is the total number of the actual *S* and *A* obtained from 1 − *I*_*k*_ + *R*_*k*_, where *ϕ*_4__*k*_^*T*^ = [−*A*_*k*_] and *θ*_4_ = [*α*]. Finally,

(13) $$\begin{array}{c} \theta_{4}={\left(\Phi_{4}^{T}\Phi_{4}\right)}^{-1}\Phi_{4}^{T}\left(S+A\right), \end{array}$$

where $\Phi_{4}={\left[\begin{array}{ccc} \phi_{4_{1}}^{T} & \phi_{4_{2}}^{T} & \phi_{4_{N_{T}}}^{T} \end{array}\right]}^{T}$ and

(14) $$\begin{array}{c} S+A={\left[\begin{array}{ccc} {{\left(S+A\right)}_{\text{fit}}}_{1} & {{\left(S+A\right)}_{\text{fit}}}_{2}\cdots & {{\left(S+A\right)}_{\text{fit}}}_{N_{T}} \end{array}\right]}^{T}. \end{array}$$

Also, according to Equation (8e),

(15) $$\begin{array}{c} I_{k-k_{d}}=\frac{1}{g}R_{k+1}. \end{array}$$

Since (1/*g*)*R*_*k*+1_ is determined, so the value of *I*_*k*−*k*_*d*__ is determined, too. Also, the value of *I*_*k*_ is known, and according to Table 1, data analysis, and comparing the differences between these two vectors, *k*_*d*_ can be obtained approximately. Now, considering the two new groups (*A*) and the delay, similarly, the unknown parameters including *β*, *g*, *μ*_*d*_, *k*_*d*_, and *α* are estimated and the new model is obtained. The uncertainty range of the estimated parameters is also presented in Table 4. The uncertainty range of the estimated parameters is also presented in Table 5.

**Figure 10:**
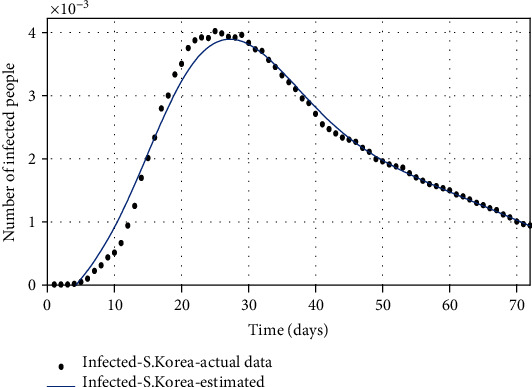
The comparison of the number of infected individuals based on the actual data and the model obtained from the estimated parameters.

**Figure 11:**
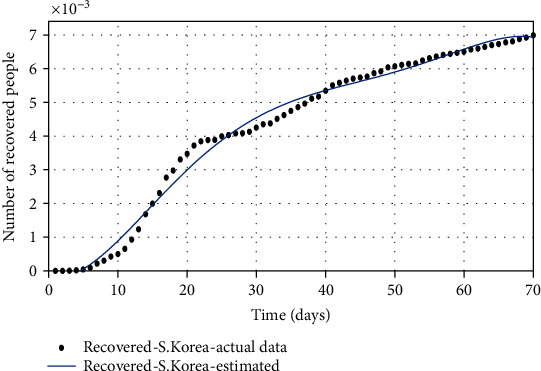
The comparison of the number of recovered individuals based on the actual data and the model obtained from the estimated parameters.

**Figure 12:**
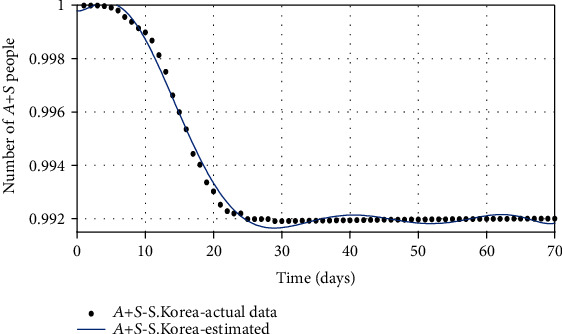
The comparison of the total number of two groups (*A* and *S*) based on the actual data and the model obtained from the estimated parameters.

Figures 10–12 show the comparison of the number of infected, recovered, and the sum of two *S* and *A* groups, respectively, based on the actual data and the model obtained from the estimated parameters.

**Figure 13:**
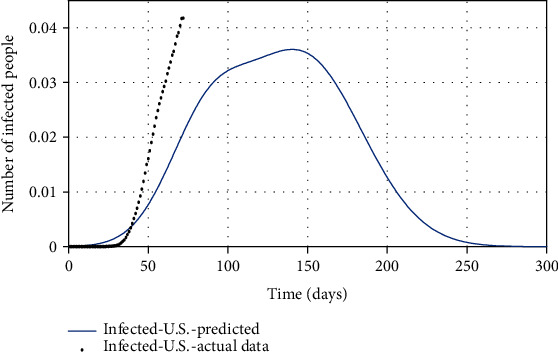
The actual number of infected people compared with the predicted number of them.

**Figure 14:**
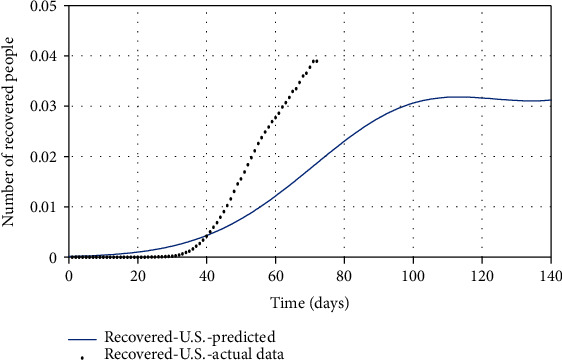
The actual number of recovered people compared with the predicted number of them.

**Figure 15:**
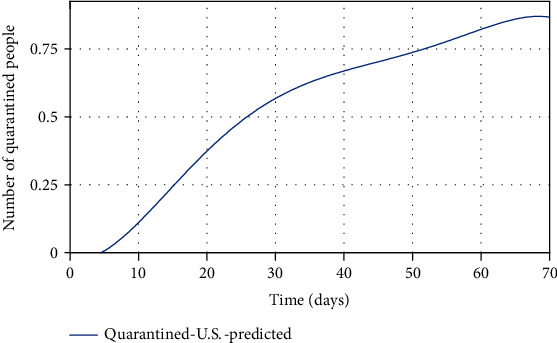
The predicted number of people in quarantine (*Q*).

Finally, by estimating *α* and *k*_*d*_, and for *ψ* = 0.95, the spread of COVID-19 in the United States can be predicted in Figures 13–15. In Figure 13, the actual data published by CDC of the United States from Feb.15 to Apr.28 are marked in black spots, and the predicted number of infected people is shown in the blue line. If 95% of susceptible people (group *S*) were quarantined from the beginning of the disease, based on this study estimation, the epidemic of COVID-19 in the United States would end within approximately seven months, and although the population has almost tripled, the peak of the disease would not increase so much (black spots in Figure 13). The possible turning point of COVID-19 epidemic in the United States will be at the end of November 2020. But even in the current situation, by applying this technique to the epidemic situation in the U.S., it can be conjectured that the eventual eradication is reached in seven months that maximizes the number of individuals who escape infection altogether.

As shown in Figure 14, there is a significant difference between the number of predicted recovered people using the proposed model and the real number of recovered people in the United States. Since in the proposed model 95% of people have been quarantined at the beginning of the disease outbreak, fewer people get infected. As a result, fewer people will be recovered from the disease, and fewer recovered people imply that convergence toward immunity will be faster, whereas the number of recovered people in the U.S. is on the rise, indicating the high number of infected people. If the United States had followed this study procedure to quarantine in early severely, then the number of recovered people would have been smaller (because there were fewer infected). In Table 6, there is no statistic of the number of asymptomatic people and susceptible people. However, based on the proposed model, it can be predicted that the number of (*A* + *S*) reached zero (Figure 9), but since the number of people is a positive number and the sum of them reached zero, it means both *S* and *A* reach zero. Achieving zero number of susceptible people means that people's quarantine is well done and the number of asymptomatic people has fallen to zero (meaning that all of them are recovered). Eventually, in Figure 15, the number of people in quarantine is demonstrated, which as expected the susceptible people are quarantined well. Although there is a lack of actual data for some groups (*A* and *Q*), the SQAIR model with estimated parameters should help forecast the epidemic of COVID-19 and prevent the spread of similar viruses in the future, since the 1918 influenza outbreak or the “Spanish flu” spread over the world (between 1918 and 1919) and about 500 million people became infected with this virus and the number of deaths estimated at least 50 million, with about 675,000 in the United States. Therefore, these simulation results are also useful not only for the first peaks but also for predicting the second peaks of COVID-19 observed in some countries or are expected soon. Accordingly, it can be estimated that the pandemic will peak during the second wave, in the fall of 2020. Hence, if the quarantine is not done correctly and is broken for any reason, it is possible to create the highly fatal next waves, as what happened in the Spanish flu in 1918. It should also be pointed out that the spreading out of COVID-19 in the United States would still be very severe. In addition to the high growth and even the mortality rate of the COVID-19 outbreak, the economic and social costs are the next problem, which are affected by this disease, and if the quarantine of people is not emphasized, it will have many catastrophic economic consequences discussed in [22]. Therefore, the law can contribute to preventing COVID-19 by supporting access to treatment and allowing public health authorities to limit contact with infectious people in response to disease outbreaks. Hereupon, the government should intervene to reduce the number of involved people, and it requires imposing martial law to strictly quarantine the population, efforts to treat infected people, and clinical research. Also, criminal penalties for breaking the quarantine and transmission of COVID-19 may create disincentives for individuals to stay home. Encouraging people to observe self-protection (like wearing a face mask, social distancing, and limiting gathering) is significant to break the transmission chain, especially in countries where rates of COVID-19 are high.

## 4. Conclusion

In this paper, the mentioned method's efficiency to identify the unknown parameters of two basic (SIR) and extended (SQAIR) epidemic models was evaluated. In this regard, first, the SIR-type model with unknown parameters was considered to investigate the dynamic of COVID-19. After that, based on the real data from the Korea Disease Control and Prevention Agency (KDCA), the unknown model parameters were estimated to predict the spreading process of COVID-19 in the United States. In the absence of effective vaccine and treatment, the number of COVID-19 infected people rises rapidly. Therefore, it is essential to consider the quarantine strategy of susceptible people to apply adequate control and decrease the risk of virus spread. Thus, by adding the new group called quarantined people, the model got more realistic. In this way, the SQIR model with unknown parameters was introduced to analyze the epidemic of COVID-19. In the following, by incorporating the impact of asymptomatic people (*A*) on the epidemic procedure, which is almost impossible to distinguish such people in society, the model developed to SQAIR model, the COVID-19 dynamic was correctly modeled. The model parameters and the delay considered for the complete recovery of the infected people were also regarded as unknown parameters in the model. In both strategies, unknown parameters of the model were estimated using real data obtained from KDCA and the least-squares method. This model was then compared with the United States' actual data published from the Centers for Disease Control and Prevention (CDC) and the possible end point of the disease, and its inflection point was predicted. Finally, the results were compared in the form of graphs. Although many countries try to break the transmission chain, traveling continues the increase of the COVID-19 prevalence. Therefore, the connections between cities and countries as network-based issues and their impact on the final result of the estimation can also be examined in future works. After developing the vaccine, the disease spread control by injecting the right amount of drug dosage at the correct times can also be an important study for future studies.

## Figures and Tables

**Table 1:** The daily number of infected people and deaths for COVID-19 in South Korea from Feb. 15 to Apr. 28, 2020 [18, 19].

*k*	Infected people (*I*_*k*_)	Daily deaths (*d*_*k*_)	*k*	Infected people (*I*_*k*_)	Daily deaths (*d*_*k*_)	*k*	Infected people (*I*_*k*_)	Daily deaths (*d*_*k*_)
0	19	0	26	7293	6	52	3445	6
1	20	0	27	7198	1	53	3408	8
2	20	0	28	7180	5	54	3246	4
3	19	0	29	7253	3	55	3125	4
4	42	0	30	7024	0	56	3026	3
5	94	1	31	6838	6	57	2930	3
6	190	1	32	6789	3	58	2873	3
7	416	0	33	6527	7	59	2808	5
8	578	4	34	6325	3	60	2750	3
9	803	2	35	6085	8	61	2627	4
10	944	3	36	5884	2	62	2576	1
11	1225	1	37	5684	7	63	2484	2
12	1729	1	38	5410	9	64	2385	2
13	2297	3	39	5281	6	65	2324	2
14	3109	1	40	4966	5	66	2233	1
15	3685	4	41	4665	8	67	2179	1
16	4277	7	42	4523	5	68	2050	2
17	5120	4	43	4398	8	69	1967	0
18	5498	3	44	4275	6	70	1843	2
19	6107	7	45	4216	4	71	1769	2
20	6415	1	46	4155	3	72	1731	1
21	6875	5	47	3979	4	73	—	—
22	7097	2	48	3867	5	74	—	—
23	7178	3	49	3654	3	75	—	—
24	7165	7	50	3591	6	76	—	—
25	7362	0	51	3500	3	77	—	—

**Table 2:** The goodness of fit criteria.

State	*S*	*I*	*R*
Criterion			
Sum of squared estimate of errors (SSE)	2.29*e* − 6	9.32*e* − 6	9.33*e* − 6
Root mean square error (RMSE)	2.08*e* − 4	2.11*e* − 4	2.12*e* − 4

**Table 3:** The uncertainty range of the parameters.

Parameter	*β*	*g*	*μ*_*d*_
Value			
Uncertainty interval	(0.993 1.018)	(0.221 0.225)	(0.021 0.0312)
Mean	~1	0.223	0.0261

**Table 4:** The goodness of fit criteria.

State	*S* + *A*	*I*	*R*
Criterion			
Sum of squared estimate of errors (SSE)	2.31*e* − 6	9.32*e* − 6	9.34*e* − 6
Root mean square error (RMSE)	2.10*e* − 4	2.11*e* − 4	2.13*e* − 4

**Table 5:** The uncertainty range of the parameters.

Parameter	*β*	*g*	*μ*_*d*_	*α*
Value				
Uncertainty interval	(0.981 1.027)	(0.217 0.228)	(0.0191 0.0324)	(0.208 0.221)
Mean	~1	0.222	0.0257	0.214

**Table 6:** The daily number of infected people and deaths for COVID-19 in the United States from Feb. 15 to Apr. 28, 2020 [19, 20].

*k*	Infected people (*I*_*k*_)	Daily deaths (*d*_*k*_)	*k*	Infected people (*I*_*k*_)	Daily deaths (*d*_*k*_)	*k*	Infected people (*I*_*k*_)	Daily deaths (*d*_*k*_)
0	12	0	26	1581	0	52	371824	2228
1	12	0	27	2126	0	53	400987	2165
2	12	0	28	2664	10	54	429785	2111
3	12	0	29	3484	15	55	460252	2236
4	12	0	30	4434	22	56	485427	2024
5	10	0	31	6129	26	57	509285	1727
6	29	0	32	9032	50	58	530230	1726
7	29	0	33	13548	68	59	553052	2566
8	28	0	34	19092	70	60	571061	2631
9	48	0	35	23870	65	61	590041	2193
10	51	0	36	33150	135	62	616864	2543
11	54	0	37	43199	180	63	636301	1883
12	54	0	38	54044	268	64	657926	1570
13	57	0	39	67231	303	65	682903	1952
14	60	0	40	82872	354	66	695770	2683
15	65	0	41	100548	496	67	722441	2358
16	85	0	42	118766	644	68	750118	2340
17	106	0	43	137133	497	69	762609	1957
18	138	0	44	158563	815	70	788233	2065
19	200	0	45	180900	1085	71	812966	1157
20	289	0	46	204966	1243	72	814569	1384
21	401	0	47	232646	1182	73	—	—
22	504	0	48	262257	1263	74	—	—
23	663	0	49	292687	1545	75	—	—
24	949	0	50	313879	1409	76	—	—
25	1248	0	51	342203	1505	77	—	—

## Data Availability

The data used are included in the paper and cited accordingly.
